# A semi-supervised learning approach with consistency regularization for tumor histopathological images analysis

**DOI:** 10.3389/fonc.2022.1044026

**Published:** 2023-01-09

**Authors:** Yanyun Jiang, Xiaodan Sui, Yanhui Ding, Wei Xiao, Yuanjie Zheng, Yongxin Zhang

**Affiliations:** ^1^ School of Mathematics and Statistics, Shandong Normal University, Jinan, China; ^2^ Shandong Provincial Hospital, Shandong University, Jinan, China

**Keywords:** deep learning, semi-supervised learning, data augmentation, consistency regularizaton, whole-slide images

## Abstract

**Introduction:**

Manual inspection of histopathological images is important in clinical cancer diagnosis. Pathologists implement pathological diagnosis and prognostic evaluation through the microscopic examination of histopathological slices. This entire process is time-consuming, laborious, and challenging for pathologists. The modern use of whole-slide imaging, which scans histopathology slides to digital slices, and analysis using computer-aided diagnosis is an essential problem.

**Methods:**

To solve the problem of difficult labeling of histopathological data, and improve the flexibility of histopathological analysis in clinical applications, we herein propose a semi-supervised learning algorithm coupled with consistency regularization strategy, called“Semi- supervised Histopathology Analysis Network”(Semi-His-Net), for automated normal-versus-tumor and subtype classifications. Specifically, when inputted disturbing versions of the same image, the model should predict similar outputs. Based on this, the model itself can assign artificial labels to unlabeled data for subsequent model training, thereby effectively reducing the labeled data required for training.

**Results:**

Our Semi-His-Net is able to classify patches from breast cancer histopathological images into normal tissue and three other different tumor subtypes, achieving an accuracy was 90%. The average AUC of cross-classification between tumors reached 0.893.

**Discussion:**

To overcome the limitations of visual inspection by pathologists for histopathology images, such as long time and low repeatability, we have developed a deep learning-based framework (Semi-His-Net) for automatic classification subdivision of the subtypes contained in the whole pathological images. This learning-based framework has great potential to improve the efficiency and repeatability of histopathological image diagnosis.

## 1 Introduction

Normal vs. tumor and cancer subtype classification *via* pathological examination is a key process in the diagnosis of cancer malignancy and treatment selection. Clinically, pathologists need to quickly and accurately analyze their patient’s biopsy and draw a pathological diagnosis report. During the diagnosis process, due to the large size of a slice, pathologists need to continuously zoom in and out of the field of view for observation to determine the key regions for diagnosis and perform classification based on features. Manual analysis of pathological slices is extremely time-consuming and labor-intensive, and some critical diagnostic information may be missed (1). In addition, the existence of difficult or ambiguous cases in pathology has aggravated the subjectivity and randomness of pathological diagnosis, resulting in inconsistent diagnoses by multiple diagnoses or different experts (2). The latest reports indicate that the clinical need for pathological analysis is increasing, and while skilled pathologists are in shortage (3).

Automated classification of tumor subtypes has become an active research topic since the emergence of whole-slide images (WSIs) technology (4, 5). Computer-aided diagnosis (CAD) systems are computer-based systems that evaluate and quantify aberrant cells and tissues in a short time, thereby helping enhance the accuracy of pathological decisions and relieving the workload of pathologists (6). Recently, there have been significant progresses in deeplearning methods for clinical analysis and research on WSIs (7), and large-scale data collection and analysis can reveal the spatial behavior shared between cancers (8).

**Figure 1:**
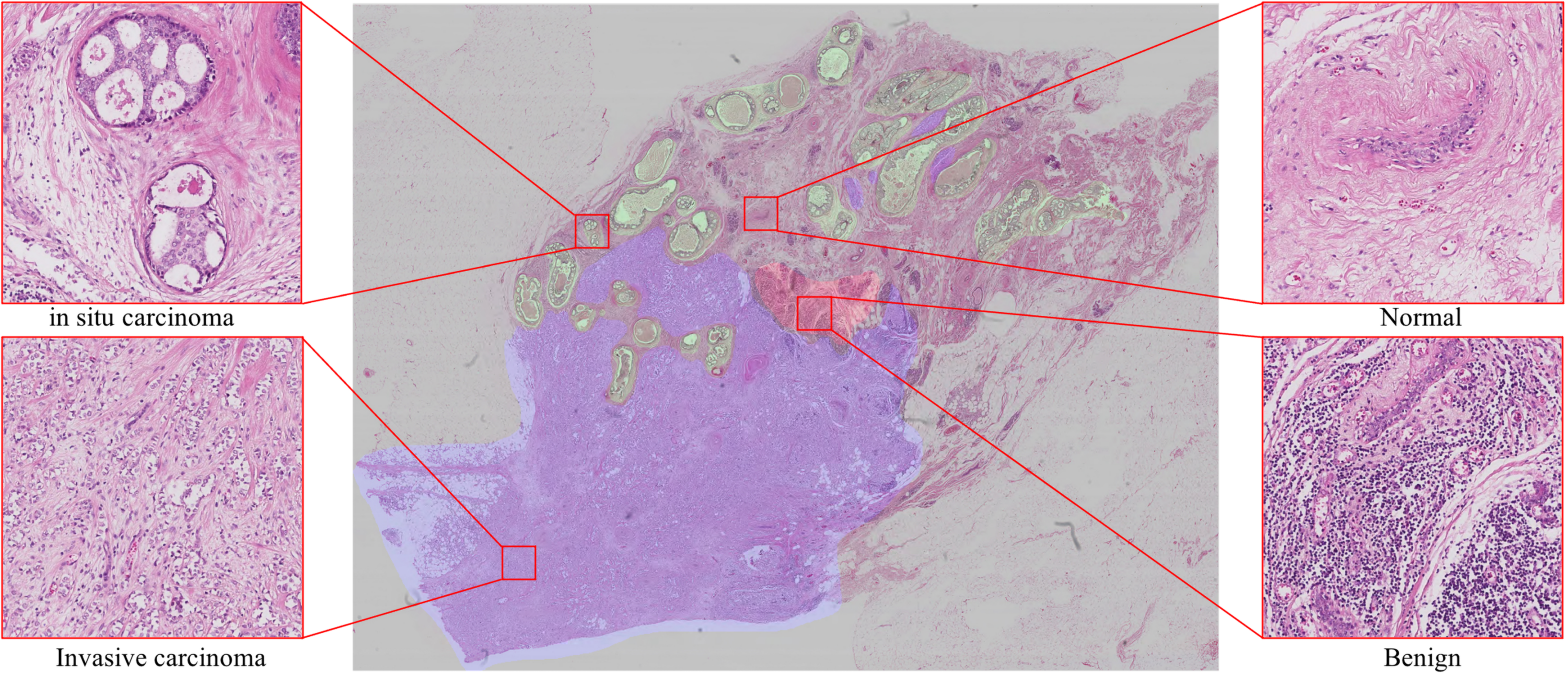
Examples show four classes in breast histology microscopic images: normal, benign, *in situ* carcinoma and invasive carcinoma.

However, due to the shortage of current computing resources, it is not feasible to use WSIs as the input of convolutional neural networks (CNNs) classification model (9) or fully convolutional networks (FCNs) segmentation model (10) to realize image analysis. One feasible scheme is to down-sample the original image to a lower resolution, which will inevitably lead to a reduction in the final accuracy. Another possible scheme is to tile the WSIs to patches for analysis and combine the results of tile analysis (7). In order to keep the accuracy of the pathological slice analysis, we adopt the second scheme to realize the whole image analysis. Deep learning approaches to WSIs analysis suffer three major limitations: 1) The labeling data of histopathological images are particularly rare. The size of WSIs is large and requires experienced pathologists to use special labeling tools and spend considerable time and cost to annotate. Deep learning-based algorithms typically require a large amount of data to perform optimal training and be able to generalize, making the model prohibitively expensive when training the model or migrating to new medical tasks (11–13). 2) The presentation of the histopathological slide is closely related to the preparation of samples (flash-frozen or formalin-fixed paraffin-embedded), and is also affected by the staining conditions, which limits the system prediction accuracy. 3) Pathological images contain a wealth of information, as shown in **Figure 1**, there are background areas (blood vessels, lymphocytes, among others) that affect the analysis.

To address the above challenges, we design to apply semi-supervised learning (SSL) to work out the normal vs. tumor and cancer subtype classification problem. Our model draws on the strategy of generating artificial labels *via* consistency regularization and pseudo-labeling in FixMatch (14). Specifically, when the model input is the same image with different disturb, the model should correspond to the same predicted distribution. Thus, for labeled data, the model’s predictions are consistent for input images with small noise (random horizontal flips). After that, given the unlabeled data with weak enhancement as input, the model predicts the distribution, and when the maximum value of the model output reaches the threshold we set in theproposed method, a valid artificial label is generated. This label will serve as a label constraint for the model training of strong augmented images for this image.

The major contributions of our study are as follows:

1. We adopt a semi-supervised scheme to classify histopathological images, which can be applied to tumor histopathological slices analysis from various tissues and organs.
2. We propose to use consistency regularization and pseudo-labeling strategies in generating artificial labels for unlabeled images, to achieve effective use of unlabeled data.
3. Considering that there may be a large number of irrelevant regions in WSIs, we recommend adding constraints to the loss function to allow the generation of empty labels, with removing these areas during the training process according to the results predicted by the model.

## 2 Related work

### 2.1 CAD systems in tumor whole-slide images analysis

In the last decade, CAD has achieved good results in WSIs analysis (5, 15). It assists doctors in clinical decision-making by detecting, quantitatively analyzing, or visualizing relevant areas with diagnostic information. Many systems and methods have been developed for this purpose. For example, Al-kofahi et al. (16, 17) developed a semi-automatic cell nucleus segmentation system for quantitative histocytometry. The system uses a graph-cut-based binarization to extract the image foreground and detect nuclear seed points *via* multiscale Laplacian-of-Gaussian filtering, which is used to obtain initial segmentation and refined using a second graph-cuts-based algorithm. Zhang et al. (18, 19) employed hierarchical voting and a repulsive active contour to detect and segment breast microscopic cells. These cells were then subjected to real-time retrieval of images with supervised kernel hashing that encodes a high-dimensional image feature vector to only tens of binary bits hash tables. In addition, several more mature softwares have been applied in pathological image analysis tasks such as annotation, visualization, cell and tissue detection by QuPath, cell-by-cell analysis and quantification by HALO (20), and cancer vs. non-cancer analysis by e-pathologists (21).

Several learning-based methods have been developed for histopathological image analysis, such as those based on CNNs for subtype classification (22), FCNs for segmentation (23), and Mask R-CNN (24) for nuclei detection and segmentation (25). The most similar to our work is classification and mutation prediction which automatically classifies lung tumor subtypes and predicts mutations (7) by learning a parametric function using an Inception v3 architecture (26). In this study, the WSIs were tiled into non-overlapping 512 × 512-pixel patches, and they were applied as input data to feed into the Inception v3 Network,classified LUAD vs. LUSC with the area under the curve (AUC) of 0.97, and six mutated genes in LUAD with AUCs from 0.733 to 0.856. Yu et al. (27) built a CNNs to classify histopathology images using lung adenocarcinoma and lung squamous cell carcinoma WSIs in TCGA, achieving AUC > 0.935 in identifying tumor regions from whole-slide histopathology images and AUC > 0.877 in recapitulated expert pathologists’ diagnosis. Noorbakhsh et al. (11) observed that CNN can not only be used for histopathological classification but also that the classifier comparison reveals intra-slide spatial similarities, i.e., the tumor/normal CNN trained on one tissue is effective for other tissues. Despite the advances highlighting the potentiality of deep learning methods in the analysis of WSIs, most of these depend on plenty of labeled images, which is a significant disadvantage compared to our method. Our work focuses on extending SSL to histopathological classification, thereby possibly reducing the dependence of deep learning models on labeled data to some extent.

### 2.2 Semi-supervised learning

SSL is a common learning method that utilizes labeled data together with unlabeled data to strengthen a model’s performance (28, 29). Extensive work has been conducted on a variety of image classifications (14, 30–35). Lee et al. (30) first proposed using pseudo-labels to effectively use unlabeled data, i.e., using the training modelto make predictions of the category of unlabeled data to acquire pseudo-labels and then utilizing cross-entropy loss to minimize errors between prediction results and pseudo-labels. Tietz et al. (31) implemented SSL by transforming Ladder Network (36), which represents relevant invariant features by a denoising autoencoder (dAE) and a clean encoder, while Laine et al. (32) simplified and optimized the previous method to make training faster and performance better. Virtual adversarial training (33) generates adversarial Gaussian noise on the input and uses entropy minimization.

Given that such methods can learn the characteristics of datasets in limited labeled data, some researchers have considered using them for WSI analysis. For example, Myronenko et al. (37) use instance pseudo-labels strategy for WSI image analysis. Chhipa et al. (38) presented a novel self-supervised pre-training method, which learns efficient representations on histopathology medical images utilizing magnification factors. The latest semi-supervised methods (14, 34) incorporate previous research: consistency regulation, pseudo-label or label sharpening, entropy minimization, and other DA strategies, and the performance has also been significantly improved. Inspired by this novel SSL mechanism, we propose extending FixMatch (14) to WSIs analysis. Our method applies to the entire WSIs, only a limited labeled area in the WSIs, or an additional limited amount of labeled data.

Data augmentation (DA) is important in deep-learning-based image classification methods by providing a model with novel training data created by transforming the original dataset. A wide variety of DA methods have been proposed in the literature, and theycan be divided into three categories: simple augmentation methods, regional-level augmentation methods, and automatic augmentation methods. Most augmentation methods are dependent on the first category: geometric transformation such as flips, crops, affine transform, and pixel-level content transformation such as invert, noise, blur, sharpness, and contrast disturbance. Several regional-level augmentation methods, such as Cutout (39) and random erasing (40), randomly mask or modify the pixel value in an area of *N* × *N* size in the image, and thus use regularization to improve model performance. The most similar applications to our work are AutoAugment (41), Fast AutoAugment (42) and RandAugment (43), which generates novel image data from the original dataset by training a sub-network to search for the appropriate augment parameters.

## 3 Methods

In the present section, we first illustrate our proposed Semi-His-Net method to analyze pipline of histopathological images and then present the key method and loss function of Semi-His-Net. We focus on classification problems, and are committed to maximizing the effect of unlabeled data and implementing the model training in a semi-supervised manner.

### 3.1 Pipline

**Figure 2:**
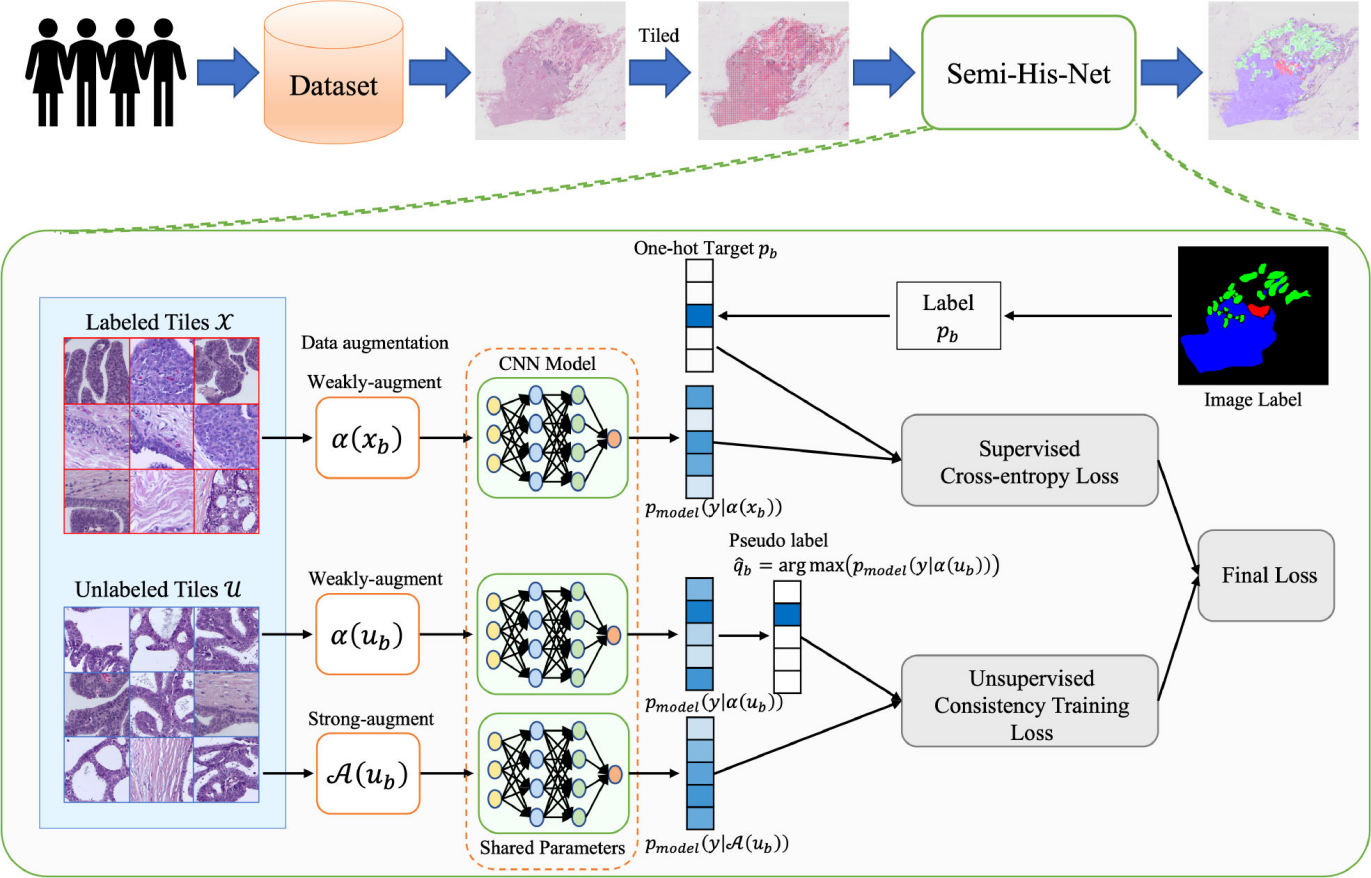
Method overview: the proposed semi-supervised histopathology image analysis method for WSIs. We learn the parameters for a CNN classification model that maps the tiled histopathology images to the tumor subtype. To make unlabeled images participate in model training, the pseudo-labels are estimated from the predicted category probability distribution of the classifier.

The architecture of Semi-His-Net is illustrated in **Figure 2**. As described above, instead of simply dividing the data into training and testing sets (the labels of the training set for the model are visible, and the labels of the testing set for the model are invisible), our method leverages partially labeled data and a considerable amount of unlabeled data. As the size of WSIs is too large and the limitation of GPU memory makes it impossible to realize the WSI convolution operation, we tiled the image into 512 × 512 pixel non-overlapping patches. We construct a semi-supervised model combining consistency regularization and pseudo-labeling, which uses a small number of labeled tiles to drive the training of the classification model. The labels of the unlabeled tiles are iteratively estimated during the training process. The following steps are included of our analysis method.

1. Organizing sample datasets. The samples are randomly divided into training and testing sets.
2. Exporting ×20 or ×40 magnification images from the original tumor, suspected tumor, or tissue normal sample slices and tile them into 512 × 512 pixel non-overlapping tiles.
3. Feeding the tiles to our semi-unsupervised model, including data with unlabeled and labeled annotations, and predicting the category of tiles. The semi-supervised model is shown in fig:framework. A schematic diagram of our model is described in sec:Semi-supervised Model.
4. Displaying the results of the model on the original WSIs.

### 3.2 Semi-supervised histopathology analysis network

In this subsection, we introduce the key models for our WSIs analysis method, a semi-supervised deep learning model based on consistency regularization and pseudo-labeling, called Semi-His-Net.

#### 3.2.1 Problem definition

There are two situations in our data: 1) the labeled part of the WSIs, and 2) some slides are manually labeled and others are unlabeled.

For an L-class classification problem, given a batch *X* of labeled data with one-hot targets and a batch *U* of unlabeled data without manual labeling. The classification model extracts features and generates the predicted class distribution *p*_*model*_(*y*|*x*) from the input image data *x*. To enable the model to capture the similarity of the effective features from the image, we calculated the cross-entropy loss CE(||) between the predicted class distribution *p*_*model*_(*y*|*α*(*x*)) generated by the model for the augmented image α (*x*) and the original one-hot label. Furthermore, for unlabeled data, the consistency regularization strategy means that the predictions of different perturbations corresponding to the same image are consistent. The model predicts the distribution from the unlabeled data and generates artificial labels as pseudo labels for its strong-augmented data *A*(*u*_*b*_) training. In this way, unlabeled data with a distribution close to labeled data can be the first to obtain pseudo-labels to participate in training, and multiple iterations to achieve predictions on the data set.

#### 3.2.2 Semi-His-Net CNN and transfer learning

The classification model is based on ResNet101 (44), which can solve the problem of deep neural network degradation and is very suitable for medical image analysis. We initialized our model with pre-trained parameters on ImageNet large dataset and then refined the parameters of the last few layers. In sec:hyperparameter, we discuss the influence of the network architecture setting and network layers optimized *via* backpropagation.

#### 3.2.3 Consistency regularization and pseudo-labeling

Consistency regularization is a commonly used method for training deep models. It relies on DA, indicating that the model should correspond to the prediction result distribution when the perturbation image of the same image is inputted (45). This type of consistency regularization method is applied in the SSL method, and it has become an important part of the latest SSL technology (32, 33, 46). Consistency regularization applied to unlabeled data relies on the assumption that the output of model will not changed when the input data is ambiguous, such a model uses unlabeled data to train the model through standard supervised classification L2-norm loss:


(1) $$\sum_{b=1}^{\mu B}p_{model}\left(y|\alpha \left(u_{b}\right)\right)-p_{model}{\left(y|\alpha \left(u_{b}\right)\right)}_{2}^{2}$$

“Pseudo-Label” (30) borrows the idea of consistency regularization in equ:1 with the model training on artificial labels to get predictive outputs on unlabeled data. This provides a simpler and more efficient strategy, and practice has proved that it can significantly improve the results. “MixMatch” (34) addresses this by sharpening the average of K prediction that use classification models to separately predict unlabeled data that undergoes Ktimes stochastic DA. In this method, we use a pseudo-label strategy to simplify the consistency regularization, so that unlabeled data learns a generated ‘‘one-hot” form of pseudo-label instead of the category probability distribution. Let *q*_*b*_=*p*_*model*_(*y*|*α*(*u*_*b*_)) be the class distribution from a given stochastic data-augmented input *α*(*u*_*b*_) through a classification model. We use ${\hat{q}}_{b}=\arg\;\max\;\left(q_{b}\right)$ to retain the maximum value of the model’s predicted distribution as a pseudo-label. The former is defined as follows:


(2) $$\frac{1}{\mu B}\sum_{b=1}^{\mu B}I\left(\max\;\left(q_{b}\right)>\beta \right)\text{CE}\left({\hat{q}}_{b}||q_{b}\right),$$

where *I*(·) is an indicator function, referring to the generation of a “one-hot” probability distribution when the maximum value of the predicted probability distribution is greater than the hyperparameter *β*. CE(||) refers to thecross-entropy between two probability distributions, ${\hat{q}}_{b}$ and *q_b_*.

In addition, because there are many normal or abnormal areas (blood cells, cytoplasm, and inflammatory cells) in WSIs that have content but are not relevant to the analysis, we recommend using zero label to effectively avoid mandatory marking of irrelevant tiles.

#### 3.2.4 Dynamic data augmentation

Motivated by the previous successful outcome of data augmentation (DA) in semi-supervised learning, we propose an optimized version of DA similar to that of RandAugment (43). Specifically, we define an image processing transformation library based on the Python Image Library that contains K transforms such as flips, affine transform, noise, blur, among others. The specific transformations are shown in **Table 1**. Subsequently, one operation in the library was randomly selected for each transformation, and *N* operations were performed. Owing to the use of different microscopes/scanners and the differences in staining schemes and chemical manufacturers, there are possible large color differences between digital histopathology images from different institutions (47). To further generalize the model during the training process, color, brightness, and other modifications were added to the image processing transformation library. Here, although a more sophisticated method can be used to select the elements in the library for combination defining an independent search algorithm to reduce the computational complexity and computational overhead, we have chosen this simpler and more effective method. The random transformation has only two parameters (the number of transformations *N* and the global distortion *M*) to guide the image processing; however, owing to the random combination, there are *N* × *K* × *M* potential transformation strategies. The number of transformations *N* and global distortion *M* are adjusted dynamically according to the number of iterations.

**Table 1:** Transformations available in Semi-His-Net.

Transformation	Description	Parameter	Range
Rotate	Rotates the picture by degrees.	*θ*	[-20, 20]
Fliplr	Flips the picture horizontally with a probability of *Λ*.	*Λ*	[0, 1]
Flipud	Flips the picture vertically with a probability of *Λ*.	*Λ*	[0, 1]
Brightness	Converts the picture to a colorspace with a brightness-related channel, adds between *−α* and *α*, then converts back to the input colorspace (RGB).	*α*	[-30, 30]
Contrast	Adjusts contrast by scaling each pixel to 127 + α*(v−127).	*α*	[0.1, 1.9]
Cutout	Fills one or more random rectangular patches in the picture using a fill mode.	*n*	[1, 5]
Dropout	Sets *p* percent of pixels in picture to zero.	*p*	[0, 0.1]
Sharpness	Sharpens the picture and covers the results with the initial picture with a strength of *Λ*.	*Λ*	[0, 1]
Shear x	Shears the picture alongside the horizontal axis in rate *S*.	*S*	[-0.3, 0.3]
Shear y	Shears the picture alongside the vertical axis in rate *S*.	S	[-0.3, 0.3]
Change Colorspace	Converts to HSV colorspace and adds a value between 0 and *α* to Hue channel, then converts back to the input colorspace (RGB).	*α*	[0, 50]
Solarize	Thresholds all pixels over value of *T*.	*T*	[0, 256]
Translate x	Transforms the picture horizontally by (Λ × image width) pixels.	*Λ*	[-0.3, 0.3]
Translate y	Transforms the picture vertically by (Λ × image high) pixels.	*Λ*	[-0.3, 0.3]
Gaussian Noise	Adds gaussian noise to the picture, sampled normal distribution on per pixel *N*(0,s).	s	[0, 0.2]
Gaussian Blur	Blurs picture using a gaussian kernel σ.	*σ*	[0, 3]
Color Temperature	Changes the temperature to Kelvin value between *α* and *β*.	α and *β*	[1000, 40000]
Invert	Inverts the image and replaces the pixels of the original image with a ratio of Λ.	*Λ*	[0, 1]

**Figure 3:**
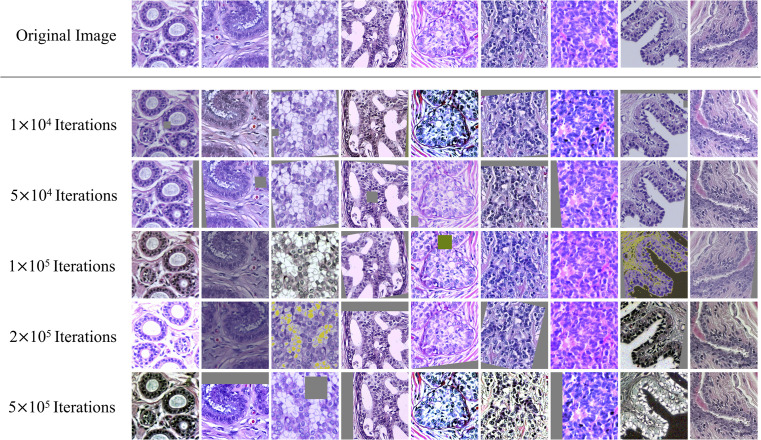
Example tiles from histopathological images, with dynamic data augmentation.

Based on this method, the disturbance is added to the tiles that are inputted into classification model. **Figure 3** displays a set of original tiles and disturbed tiles at different iteration times. In the beginning, the disturbance is not obvious, and with the training of the model, the disturbance becomes increasingly obvious.

#### 3.2.5 Loss function

There are two situations of data would be feed into the classification model, which correspond to different constraints: 1) For tiles with a one-hot label and a weakly augmented image, we use supervised cross-entropy loss. 2) For tiles without labels, we use unsupervised consistency training loss.

**Supervised cross-entropy loss:** Given a batch *X*={(*x*_*b*_,*p*_*b*_):*b*∈(1,…,*B*)} of labeled data, where the data *x_b_* with one-hot targets *p_b_*. The loss function *ℒ*_*x*_ on the labeled data is defined as:


(3) $${ℒ}_{x}=\frac{1}{B}\sum_{b=1}^{B}\text{CE}\left(p_{b}||p_{model}(y|\alpha \left(x_{b}\right)\right),$$

where CE(||) refers to the cross-entropy between two probability distributions *p_b_* and *p*_*model*_(*y*|*α*(*x*_*b*_)) , *p*_*model*_(*y*|*x*_*b*_) is the model prediction for the input *x_b_*, *α*(*x*_*b*_) is weakly-augmented data.

**Unsupervised consistency training loss:** Given a batch *U*={*u*_*b*_:*b*∈(1,…,*B*)} of unlabeled data, where the data *u_b_* without manual label. We used a pseudo-label method (30). First, for weakly-augmented data, the model predicts the class distribution *q*_*b*_=*p*_*model*_(*y*|*α*(*u*_*b*_)) . Subsequently, we use ${\hat{q}}_{b}=\arg\;\max\;\left(q_{b}\right)$ to retain the maximum value of the model’s predicted distribution as a pseudo-label, and *q_0_* to indicate that all classification distribution are 0 as a zero label. The loss function *ℒ*_*u*_ on unlabeled data is defined as:


(4) $${ℒ}_{u}=\frac{1}{\mu B}\sum_{b=1}^{\mu B}\{\begin{array}{ll} \text{CE}\left(q_{0}||p_{model}\left(y|A\left(u_{b}\right)\right)\right), & \text{if }\max\;\left(q_{b}\right)\leq \eta \\ 0, & \text{if }\eta <\max\;\left(q_{b}\right)\leq \theta \\ \text{CE}\left({\hat{q}}_{b}||p_{model}\left(y|A\left(u_{b}\right)\right)\right), & \text{if }\max\;\left(q_{b}\right)>\theta , \end{array}$$

where *A*(*u*_*b*_) is strong-augmented data. The extremely low prediction distribution (*max* (*q*_*b*_)≤*η* ) is constrained by setting the cross-entropy loss between prediction distribution and all-zero distribution to reduce the impact of potentially uncertain tiles on the currently labeled tiles for the classification model. The “one-hot” probability distributions valid when the maximum value of the predicted probability distribution is greater than the hyperparameter *θ*.

The two losses are then summed for the full objective loss *ℒ*_*Total*_ :


(5) $${ℒ}_{Total}={ℒ}_{x}+\lambda {ℒ}_{u}.$$

when the labeled tiles and unlabeled tiles are mixed for training, we use the weighting factor *Λ* to equalize the supervised/unsupervised loss.

## 4 Experiments and results

In this section, we evaluate the efficacy of Semi-His-Net in the WSI analysis. We relied on two public histopathology image datasets: BACH and TCGA.

### 4.1 Implementation details

Our Semi-His-Net was implemented using PyTorch^1^ and run on two NVIDIA A100 Tensor Core GPUs. We used the optimizer of SGD with Nesterov Momentum, which has a momentum of 0.9. And the batch size is 32. The learning rate is initialized to 0.01 and divided by 10 every 10 epochs. We evaluated models using an exponential moving average with a decay of 0.999 and applied a weight decay of 0.0004. In addition, in all experiments, we take advantage of weak augmentation on the labeled data, i.e., random horizontal flipping, and we use strong enhancement strategy for the unlabeled data, i.e., the augmentation of the random magnitude (43) in the augmentation library. In the training process, we used 3-fold cross-validation on the training set to train the models in the three experiments.

### 4.2 Experiments on ICIAR 2018 breast cancer histology image dataset

#### 4.2.1 Datasets

The breast cancer histology image datasets were acquired from the challenge on BACH^2^. The challenge contains two goals: Task 1 is to automatically divide the breast histological microscopic images stained by hematoxylin and eosin (H&E) into four subtypes (normal tissue, benign abnormality, malignant carcinoma in situ, and malignant invasive carcinoma). This task corresponds to 400 RGB color microscope images (There are 100 images in each of the four subtypes.) with a size of 2048 ×1536 pixels and pixel scale 0.42*μm* ×0.42*μm*. Task 2 is to locate the lesion areas including benign, situ carcinoma, and invasive carcinoma subtypes in the WSIs. This task corresponds to 30 WSIs witch have pixel-scale of 0,467 *μm*/*pixel*. Among them, 10 WSIs are labeled pixel-wise and 20 are unlabeled.

### 4.2.2 Contribution of unlabeled data

The microscopic image dataset was randomly assigned to two sets: a training set (80%) and a test set (20%). All images were re-divided into 512 × 512 patches with a step size of 256 pixels; that is, each original image corresponded to 35 new patches. Each patch corresponds to the labels of the predominant cancer types, and these patches constitute the labeled data in the training set. A total of 20 unlabeled WSIs in the WSI dataset were tiled by non-overlapping 512-×512-pixel windows and retained those tiles with the foreground area over 50% as the unlabeled data in the training set.

We compared the proposed Semi-His-Net with the classic CNN classification methods. In sec:Problem Definition, we introduced the definition of this method, that is, the classification model can learn from unlabeled data to alleviate the demand for labeled data.

Each microscopy image in the test dataset was divided into 35 patches, and each patch was fed through the CNN model to predict the subtype. Subsequently, the average 35 prediction results are used to generate one-hot prediction by adopting the largest distribution. As for the evaluation metrics, we utilize precision and recall to measure the accuracy of classification


(6) $$precision=\frac{TP}{TP+FP},$$

(7) $$recall=\frac{TP}{TP+FN},$$

where *T* and *F* denote the correct or not, *P* and *N* denote positive and negative. *TP* and *FN* denote the positive class prediction is positive and positive class is predicted as negative, and *FP* and *TN* denote the negative class is predicted as positive and negative class prediction is negative, respectively.

In addition, we calculated the accuracy of four subtypes. For multi-classification problems, the accuracy is the same as the results of F1-score, Micro-precision and Micro-recall, defined as:


(8) $$Accuracy=\frac{TP+TN}{TP+TN+FP+FN}.$$

The accuracy, precision and recall of these classification methods were listed in **Table 2**, which includes classic fully supervised deep learning models and the state-of-the-art semi-supervised models. After the analysis of the results of the full-supervised model, ResNet101 was used as the main backbone of the subsequent semi-supervised model. All the models are trained and tested with PyTorch on the platform of two NVIDIA A100 Tensor Core GPUs with the parameter settings: mini-batch size (32), learning rate (initialized to 0.01 and divided by 10 every 10 epochs), momentum (0.9), weight decay (exponential moving average with a decay of 0.999 and weight decay of 0.0004). Taking the fully supervised ResNet101 as the baseline, we could find that the performance of the model with the semi-supervised strategy, MixMatch, FixMatch and the proposed Semi-His-Net has been significantly improved, with the accuracy increased by 1.2%, 1.2% and 3.7% respectively.

**Table 2:** Comparison of different classification methods.

			Normal		Benign		*in situ* carcinoma		Invasive carcinoma	
Model		Accuracy	P	R	P	R	P	R	P	R
Supervised	VGG (9)	56.3 ( ± 10.9)	60.0	60.0	61.1	55.0	60.0	60.0	62.5	45.5
	Inception (26)	71.3 ( ± 9.9)	68.4	65.0	71.4	75.0	68.1	75.0	66.7	77.8
	ResNet101 (44)	86.3 ( ± 7.5)	89.4	85.0	81.8	90.0	80.9	85.0	94.4	85.0
Semi-supervised	Pseudo-Labeling (30)	61.3 ( ± 10.7)	57.9	55.0	61.9	65.0	52.4	55.0	73.7	70.0
	Mean Teacher (48)	70.0 ( ± 10.0)	66.7	70.0	68.4	65.0	70.0	70.0	75.0	75.0
	MixMatch (49)	87.5 ( ± 7.2)	90.0	90.0	80.9	85.0	85.0	85.0	94.7	90.0
	FixMatch (14)	87.5 ( ± 7.2)	94.4	85.0	85.0	85.0	81.8	90.0	90.0	90.0
	Semi-His-Net	**90.0 (**± **6.6)**	90.0	90.0	89.4	85.0	85.7	90.0	95.0	95.0

We show the average Accuracy with a 95% confidence interval in parentheses, Precision (P) and recall (R) (%) of four subtypes: normal tissue, benign abnormality, malignant carcinoma in situ, and malignant invasive carcinoma. Bold font indicates best result obtained for predictions.

#### 4.2.3 Interactive guided learning

To evaluate the proposed Semi-His-Net algorithm on the WSI dataset, we compared its semi-automated analysis results guided by label data with the model analysis results of the supervised training model. For the supervised method, we used the trained ResNet-101 model introduced in the previous section to test the WSI images directly. The labeled data of semi-automated analysis was obtained by the microscopic image dataset and partial area from the WSI dataset. When Semi-His-Net was used in WSI images, the tiles from the WSIs could as unlabeled data, and they could be used with labeled data to optimize the classification model further iteratively; they could also select a part of the area on the WSI image (manual labels corresponding to WSI images from WSI dataset, simulating pathologists marking typical areas on the WSIs images) with the patches from the microscopic image dataset as label data to realize interactive learning.

**Figure 4:**
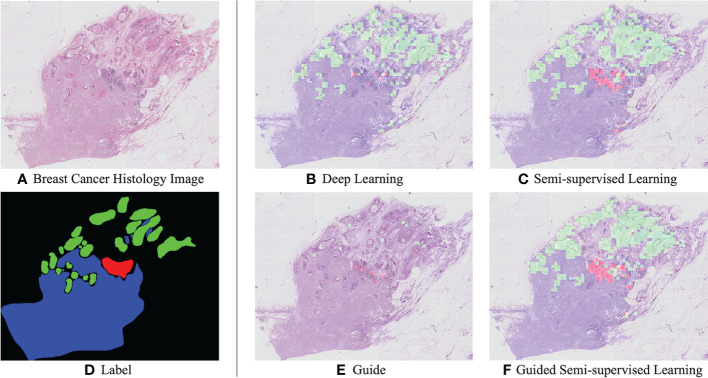
Example histological images of breast cancer **(A)** and corresponding manully label **(D)**. Results of breast cancer analysis: A visual comparison of the supervised ResNet-101 model **(B)** and the semi supervised method **(C)**. In particular, **(E)** is to randomly select some tiles from WSI images as marking data, that is, to simulate pathologists to mark typical areas. **(F)** is the result of interactive guided semi-supervised learning.

Examples of semi-automated analysis results are shown in **Figure 4**. The prediction results considerably improve with the use of SSL by comparing **Figures 4B**, **C**. Randomly select some tiles from the WSI images to associate with the ground truth labels, as shown in **Figure 4E**, to increase the proportion of label data in the training set; simultaneously, it could simulate pathologists that label typical areas to achieve semi-automatic analysis of WSI images. Then, the interactive training based on the private annotation data can improve the quality, and the comparison result images **Figures 4C**, **F** can be observed.

### 4.3 Experiments on TCGA dataset

#### 4.3.1 Datasets

To validate the effectiveness of SSL in transfer learning, 11 tissues of cancer histopathological images were obtained from TCGA, including lung cancer, kidney cancer, gynecological cancer, and gastrointestinal cancer. Specifically, lung cancer data includes two major histologic types of non-small cell lung cancer: adenocarcinoma (LUAD) and squamous cell carcinoma (LUSC); Kidney cancer data includes three subtypes: clear cell (KIRC), papillary (KIRP), and chromophobe (KICH). Gynecologic cancer refers to the cancer that starts in woman’s reproductive organs, data in this work includes three subtypes: uterine corpus endometrial carcinoma (UCEC), breast invasive carcinoma (BRCA), ovarian serous cystadenocarcinoma (OV). Gastrointestinal cancer refers to the cancer that starts from the gastrointestinal tract and accessory organs of digestion, data in this work includes three subtypes: colon adenocarcinoma (COAD), rectum adenocarcinoma (READ), stomach adenocarcinoma (STAD).

#### 4.3.2 Shared representation across tumor types

The cross-classification strategy was used to verify the spatial relations between different tumor types. Specifically, we used all WSI images in each subtype to train a CNN classifier model to distinguish normal/tumor tissues and used the trained model to predict normal/tumor slices of other cancer types. Interestingly, the average AUC of cross-classification reached 0.893 (AUC of 0.88 ± 0.11, in Ref. Noorbakhsh etal. (11), the tumor data sample selected herein is not completely consistent with this work.). The feasibility of cross-classification proves that there are shared morphological features between cancer types.

**Figure 5:**
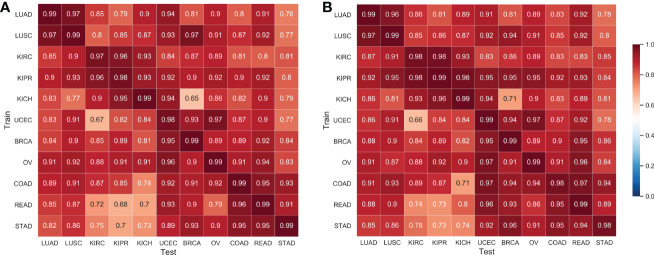
AUC of cross-classifications on eleven tissues by the supervised CNN classification model and the proposed semi-supervised method. **(A)** Results by the supervised CNN classification model; **(B)** Results by the proposed semi-supervised method. The horizontal axis of the heatmap represents the classification model trained on one set of cancer type data, and the vertical axis represents the AUC performance of the test data on the corresponding training data.

We applied the proposed Semi-His-Net for cross-classification. All WSI images in one type were selected as labeled data, and the rest of the cancer type data were used as unlabeled data to participate in the training analysis of the classification model. **Figure 5** shows the AUC using the supervised CNN classification model and the proposed semi-supervised method. The Semi-His-Net model has improved AUC in the cross-analysis of most tumors compared to the fully-supervised model, thereby also reflecting that the SSL strategy is more conducive to discovering the hidden spatial relationships between histopathological from different tumor types.

### 4.4 Network architectures and hyperparameter settings

#### 4.4.1 Influence of backbone architectures

We investigated the impact of the network architecture on the performance of Semi-His-Net. Especially, we use the Inception v3 architecture (26), ResNet (ResNet18, ResNet34, ResNet50, ResNet101, ResNet152) (44), and DenseNet (DenseNet121, DenseNet161, DenseNet201) (50) as the state-of-the-art image classification algorithms. To distinguish between lung adenocarcinoma and squamous cell carcinoma on TCGA-LUAD and TCGA-LUSC datasets by histopathological image, we replaced only the backbone network used for classification, and reported the performance of subtype classification achieved by these backbones in tab:dis1. The dataset consists of 956 histopathological slides from 956 patients.Here, only diagnostic slides from the dataset were selected. The dataset is divided randomly into training set and the test set by 8:2.

**Table 3** shows that ResNet101 backbone achieves slightly better results in classifying the four tissues. Experimental results show that, regardless of whether it is DenseNet or ResNet, a deeper network indicates better analysis results, which has also been verified in other image classification or segmentation tasks Khened et al. (51) Cheng et al. (52).

**Table 3:** Area Under the Curve (AUC) achieved by different models (%).

Model	Normal	LUAD	LUSC
Inception v3	0.990	0.956	0.966
ResNet18	0.979	0.966	0.971
ResNet34	0.984	0.967	0.973
ResNet50	0.988	0.969	0.973
ResNet101	**0.992**	**0.972**	**0.974**
ResNet152	0.991	0.969	**0.974**
DenseNet121	0.990	0.970	0.969
DenseNet169	0.983	0.968	0.970
DenseNet201	0.991	0.970	0.971
DenseNet161	0.983	0.969	0.971

Bold font indicates best result obtained for predictions.

#### 4.4.2 Influence of hyperparameters

**Figure 6:**
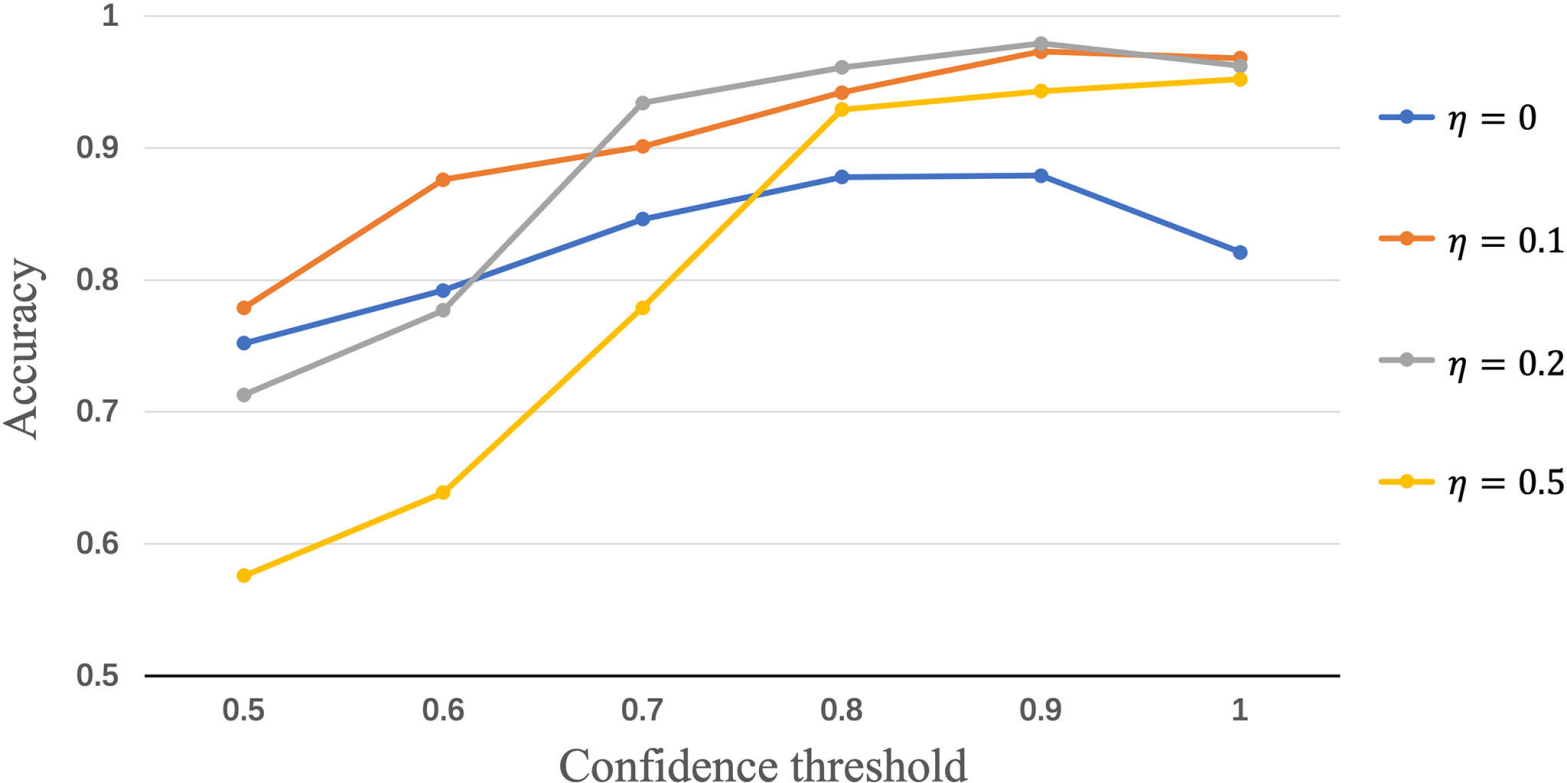
Measuring the effect of varying the confidence threshold values η and θ.

To evaluate the influence of the confidence thresholds *η* and *θ*, we show the performance achieved by Semi-His-Net with different threshold values. In the above experiments, we set four fixed *η* values to compare the effect of *θ* on the model performance. The results of accuracy score are presented in **Figure 6**, where the threshold values of *θ* = 0.9 and *η* = 0.2 show the highest accuracy. When *η* is fixed, the result will increase with an increase in *θ*, the highest AUC is displayed at the threshold value of 0.9, and further increases the threshold value *θ*, and the AUC will not be increased. Here, the threshold value *η* controls the quality and quantity of pseudo-labels; that is, the accuracy of pseudo-labels increases with the increase of the threshold *θ*, which directly affects whether the unlabeled data in training with pseudo-labels, thus affecting the contribution unlabeled loss function *ℒ*_*u*_.

## 5 Discussion

In this study, we developed a semi-supervised deep-learning analytical framework that can automatically and efficiently differentiate the subtypes of tumor pathology. We know this is not complicated work, but a good combination of the semi-supervised deeplearning method and H&E staining pathological image analysis. Using simple and efficient consistency regularization and pseudo-labeling strategies, the labeled data required by the training model is reduced, and a large number of unlabeled data can be involved in the training of the model.

In addition, a variety of deep learning algorithms have been developed for automatically predicting the subtypes of tumors (7, 53, 54), such as Lu et al. (53) proposed a CLAM (clustering-constrained-attention multiple-instance learning) method to the subtyping of renal cell carcinoma and non-small-cell lung cancer as well as the detection of lymph node metastasis. This learning-based framework has two potential merits: (1) Automatic classification results predicted by the model can remove subjective deviation and ensure reproducible decisions. (2) The prediction time of a WSI is about 30s (depending on the specific size and content of the image), which greatly reduces the time required comparedwith manual evaluation, therefore, the auxiliary diagnosis process can reduce the healthcare burden. However, most studies were only evaluated for specific organs or data with specific acquisition protocols, which affects their clinical applicability in multi-center data or migration to other organs. Secondly, in addition to the BACH dataset used in our study, there have been various international challenges recently, from which we can see the clinical demand for automated pathological analysis techniques. Most of the annotations are for patches rather than the whole image. These annotations are very time-consuming and labor-intensive and have high requirements on pathologists’ professionalism and clinical experience. Therefore, such annotations are veryprecious for research.

In conclusion, to overcome the limitations of visual inspection by pathologists for histopathology images, such as long time and low repeatability, we have developed a deep learning-based framework (Semi-His-Net) for automatic classification subdivision of the subtypes contained in the whole pathological images.This learning-based framework has great potential to improve the efficiency and repeatability of histopathological image diagnosis.

## 6 Conclusion

In this study, we presented a original CNNs-based SSL framework to analysis tumor histopathological images, called Semi-His-Net. Specifically, for unlabeled images, we use consistency regularization and pseudo-labeling to encourage the same image with different perturbations to have similar distributions predicted by the model. By integrating these strategy into CNNs model, the dataset used to train the Semi-His-Net model only needs to have a small number of images containing labels and some unlabeled image, which makes training and usage more flexible and competitive with supervised CNN models. Our proposed method was evaluated by analyzing histopathological images for tumor segmentation and subtype classification. Experimental results show that our Semi-His-Net achieved the best analysis performance, and it is adapted to transfer learning owing to the spatial behavior shared between tumor types.

## Data availability statement

The original contributions presented in the study are included in the article/supplementary material. Further inquiries can be directed to the corresponding authors.

## Author contributions

All authors made substantial contributions to the manuscript. YXZ and WX developed the study concept. YJZ and YD performed the data analysis. YJ and XS completed the method and experiment, and drafted the manuscript. All authors critically revised the paper for important intellectual content. All authors have read and agreed to the published version of the manuscript.

## Footnotes

1
https://github.com/pytorch/pytorch

2
https://iciar2018-challenge.grand-challenge.org
